# Review on III-V Semiconductor Single Nanowire-Based Room Temperature Infrared Photodetectors

**DOI:** 10.3390/ma13061400

**Published:** 2020-03-19

**Authors:** Ziyuan Li, Jeffery Allen, Monica Allen, Hark Hoe Tan, Chennupati Jagadish, Lan Fu

**Affiliations:** 1 Department of Electronic Materials Engineering, Research School of Physics, The Australian National University, Canberra, ACT 2601, Australia; ziyuan.li@anu.edu.au (Z.L.); Hoe.Tan@anu.edu.au (H.H.T.); Chennupati.Jagadish@anu.edu.au (C.J.); 2 Air Force Research Laboratory, Munitions Directorate, Eglin AFB, FL 32542, USA; jeffery.allen.12@us.af.mil (J.A.); monica.allen.3@us.af.mil (M.A.); 3 Australian Research Council Centre of Excellence for Transformative Meta-Optical Systems, Department of Electronic Materials Engineering, Research School of Physics, The Australian National University, Canberra, ACT 2601, Australia

**Keywords:** III-V semiconductor, nanowire, infrared photodetector

## Abstract

Recently, III-V semiconductor nanowires have been widely explored as promising candidates for high-performance photodetectors due to their one-dimensional morphology, direct and tunable bandgap, as well as unique optical and electrical properties. Here, the recent development of III-V semiconductor-based single nanowire photodetectors for infrared photodetection is reviewed and compared, including material synthesis, representative types (under different operation principles and novel concepts), and device performance, as well as their challenges and future perspectives.

## 1. Introduction

In the last decades, there has been an increased demand for semiconductor infrared (IR) photodetectors, due to their broad defense and civilian applications such as imaging, night vision, free space optical communication, search and rescue, surveillance, missile tracking, material inspection and identification, scientific instrumentation (e.g., spectroscopy), and astronomy [1,2]. For example, the current state-of-the-art infrared photodetector technology in the market has been mainly based on the planar semiconductor detectors such as Si (0.4–1.1 µm), InGaAs (1.1–1.7 µm), Ge (0.7–1.8 µm), InAs (0.9–3.5 µm), and HgCdTe (2–10.6 µm). Although most of these detectors have been explored for decades with mature industrial platforms, limited spectral range, low responsivity, and high noise level at room temperature have limited their use in many practical applications. In particular, in narrow bandgap materials such as InAs [3], InSb [4,5], InAsSb [6], and HgCdTe [7] with extended detection wavelength from near- and short-wavelength infrared (NIR and SWIR) to mid-wavelength infrared (MWIR) and beyond, the detectivity degrades significantly at longer wavelengths due to increased dark current resulting from carrier generation–recombination and minority carrier diffusion [8]. In the planar photodetector device design, a common compromise that is often made is to reduce absorption volume to suppress the dark current while sacrificing some responsivity. Leveraging the development of nanotechnology, many nanostructures, such as nanowires (NWs) [9,10,11], nanotubes [12,13,14], nanopillars [15,16,17], nanorods [18,19,20], and two-dimensional (2D) materials [21,22,23], have emerged in the pursuit of high-performance room-temperature photodetectors, as their nanoscale size offers extremely small active volume (for reduced dark current) and at the same time, distinct light absorption properties for large responsivity, as well as shorter response time and larger bandwidth [24].

One-dimensional (1D) semiconductor nanowires have attracted significant attention as the promising candidates for the next generation nanoscale optoelectronic devices such as photodetectors [9,10,11], solar cells [25,26,27], light-emitting diodes (LEDs) [28], and lasers [29,30,31]. These devices have unique geometry and physical properties leading to excellent optical and electrical properties; quantum size effects; as well as enhanced light, biological, or chemical sensitivity [32,33]. In particular, III-V semiconductor nanowires have direct and widely tunable bandgap, high absorption coefficient and carrier mobility, as well as flexibility to form heterostructures, making them excellent candidates for photodetection [1,33]. Moreover, it is found that the enhanced photocarrier lifetime and decreased transit time in nanowire detectors lead to much higher gain in nanowires in comparison to bulk structures [34]. Researchers have also implemented transistor structures [11,35,36,37], metal-semiconductor (M-S) or metal-semiconductor-metal (M-S-M) Schottky junctions [38,39,40,41], p-n junctions [42,43], and heterostructures [42,43,44,45,46] in nanowire-based photodetectors to further reduce dark current and enhance *I*_light_/*I*_dark_ ratio for infrared photodetection. In addition, surface passivation [11,36,44] and introduction of the photo-gating effect [36,37,47] have been carried out to manipulate the surface states of the nanowire materials to further enhance their performance. It has also been demonstrated that metal-cluster decoration [11] and nanoantenna structures [48] are capable of localizing and enhancing the spectral selectivity and light absorption of nanowires by coupling of strongly resonant and highly localized plasmonic modes.

For practical applications, ensemble nanowire array photodetectors are important. However, single nanowire-based devices provide a much simpler platform for fundamental study without having to consider complex effects such as optical coupling of surrounding nanowires as well as nonuniformity effects that arise from nanowire growth and contact formation processes in ensemble devices [33]. Here, the recent development of III-V semiconductor nanowire infrared photodetectors operating at room temperature is reviewed with an emphasis on single nanowire-based photodetectors. This includes nanowire synthesis approaches and mechanisms, typical device structures and performance, as well as the current challenges and some strategies for development toward high performance future applications.

## 2. Nanowires Synthesis

III-V semiconductor nanowires can be either synthesized by top-down or bottom-up approaches, or a combination of both [33,49,50]. In the top-down method, nanowire structures are obtained through direct writing or wet/dry etching of lithographically-defined patterns on a bulk substrate or layered structure. Nanolithography techniques can be used to form ordered nanowire arrays with accurately defined placement, size, spacing, and orientation [33,49]. However, top-down methods do not offer the important advantages of material saving and flexibility in material/structure design benefiting from the effective strain relaxation as offered by bottom-up nanowire synthesis methods. Additionally, the etching process can introduce surface defects and roughness on the sidewall that adversely affect the nanowire properties and lead to degraded device performance [33,51]. Therefore, top-down approaches are rarely used for single nanowire photodetectors and have been thoroughly reviewed [33]. In this review, we mainly focus on the discussion on bottom-up approaches.

In the bottom-up method, freestanding nanowires are grown anisotropically on a substrate along the axial direction by employing their constituent atoms. Several techniques [33,50] have been explored for nanowire growth. These include metal organic [44,52] or solid-source [53] chemical vapor deposition (CVD), molecular beam epitaxy (MBE) [54], and energy transfer method such as pulsed laser ablation [55]. The bottom-up method is especially promising for future highly-integrated electronic and optical systems as high-quality nanowire growth has been demonstrated on various substrates [56,57].

The bottom-up nanowires are normally synthesized via catalyst assisted vapor–liquid–solid (VLS) [9,44,58] and/or vapor–solid–solid (VSS) [59] growth mechanisms. The catalyst can be either disparate metals such as Au nanoparticles (foreign metal-catalyzed growth) [9,44] or particles that contain constituent elements of the nanowire (self-catalyzed growth) [60]. Normally such nanowire ensembles have no ordering unless a mask is used to arrange the seed particles [56]. Site-controlled VLS growth has also been used to grow planar nanowires [61].

Non-catalyzed growth techniques, such as selective area epitaxy (SAE) [62,63], are also often used to form nanowires on a prepatterned substrate, to accurately define position, geometry, and uniformity of the nanowire arrays [33]. Oxide-assisted growth (OAG) [64] is another mechanism that is used to grow nanowires. In this method, the semiconductor and its oxide are adsorbed on the substrate surface and the semiconductor atoms create nucleation centers, which then assist the formation of the semiconductor nanowires. The oxide acts as a passivating shell that suppresses the crystal growth in the lateral direction [64]. Some research has also examined the use of template-assisted (TA) mechanisms that employ a prefabricated template such as anodic aluminum oxide (AAO) [65] membrane to control the nanowire diameter [50].

## 3. Single Nanowire Photodetectors

Due to their distinct geometry, single nanowire devices can be fabricated into two different device configurations: (i) horizontal configuration where the nanowires are transferred from free standing positions to lying on another substrate horizontally on their growth axes, and (ii) vertical configuration, where the nanowire is perpendicular to the substrate. These different configurations lead to different absorption properties [33,50] and variation in the fabrication techniques [33]. In the horizontal configuration, light is illuminated onto the nanowire in a perpendicular direction to its axis, whereas in the vertical configuration, the direction of incident light is parallel to the nanowire axis. Vertical nanowire devices have a much larger absorption cross section compared with their physical cross section, and thus a larger absorption efficiency [25]. Single vertical nanowire photodetectors, however, have been rarely reported [43,66], which could be due to practical considerations and challenges for material growth and device fabrication. In comparison, single horizontal nanowire photodetectors are more commonly implemented and characterized and have been reported widely in many material systems. In this section, typical III-V single nanowire infrared photodetectors are discussed based on their device structures and operating wavelengths. The associated performance metrics such as responsivity (*R*), specific detectivity (D^*^), gain (*G*), external quantum efficiency (*EQE*), and response time (*t*) [1,34] under room temperature are summarized and compared in Table 1.

### 3.1. Photoconductors

One of the simplest forms of photodetectors are photoconductors that produce current under an optical excitation when an external bias is applied. The current increases with the light illumination, exceeding dark current, and thus provides the light-detection ability. The geometry and nanoscale dimensions of nanowire photoconductors enable enhanced light absorption, polarization sensitivity, and internal photoconductive gain compared to bulk photoconductors [48,67,68]. For example, similar to bulk/planar devices, light at different wavelengths can be detected using nanowires based on different materials. Yang et al. demonstrated a single-nanowire spectrometer based on a compositionally engineered semiconducting CdS*_x_*Se_1-*x*_ nanowires [69]. It could reconstruct incident spectra by measuring photocurrents along the nanowire axis, indicating that single nanowire devices are potential candidates as ultracompact microspectrometers for accurate and broadband light reconstruction, as well as high-resolution spectral imaging [69]. It has also been shown that the light absorption in Ge nanowires can be spectrally tuned and enhanced by engineering their size, geometry, and orientation as the incident light can be efficiently coupled to strong resonant modes supported in such subwavelength, high refractive index semiconductor nanostructures [70]. Moreover, some III-V nanowires, such as GaAs [71] and InP [72], show ultrafast photocarrier dynamics, indicating their potential application as high-speed photodetectors.

Among III-V nanowire photoconductors, narrow bandgap binary nanowire materials, such as InAs [11], GaSb [73], and InSb [74], can provide broadband photodetection spanning from visible (VIS) to IR regions. InAs nanowires have been demonstrated to have high carrier mobility, easy formation of ohmic contact, as well as excellent optoelectronic properties [11]. Miao et al. reported a single InAs nanowire near-infrared photodetector with a detection wavelength up to ∼1.5 μm and photocurrent responsivity as high as 1.9 × 10^3^ A/W [11]. The carrier mobility and photoresponsivity could be further enhanced by addition of an Al_2_O_3_ or HfO_2_ passivation layer that can suppress the negative influence of surface defect states and atmospheric molecules [11]. Yuan et al. showed that nanowire photodetector responsivity can also be enhanced using in situ passivation by epitaxial growth of a GaInP shell on GaAs core nanowires [75], whereas Li et al. demonstrated enhanced infrared photoresponse of GaAsSb nanowires by the in situ passivation of adding an InP shell [76]. Moreover, core–shell heterostructures, such as InAs/AlSb core–shell [46], form a type-II bandgap alignment that further improves charge carrier separation, photosensivity, and photoresponse. Single GaSb nanowire photodetectors have also been fabricated on rigid SiO_2_/Si and flexible polyethylene terephthalate (PET) substrates with high responsivity and fast response coupled with stable photoswitching in a broad spectral range from ultraviolet (UV) to NIR [73].

**Figure 1:**
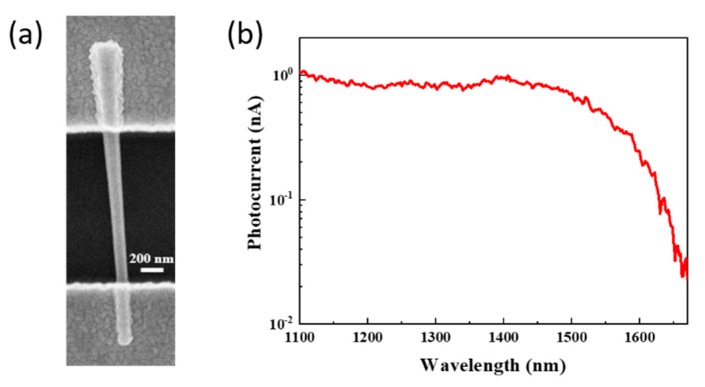
(**a**) Scanning electron microscopy (SEM) image of a single horizontal GaAs_0.56_Sb_0.44_ nanowire photodetector and (**b**) its room temperature photocurrent spectral response at a bias of 0.15 V. Reproduced with permission [9]. Copyright 2015, IOP Publishing Ltd.

Ternary III-V nanowires with tunable bandgaps, such as GaAsSb [9], InGaAs [77], and InAsP [78], have been also extensively studied. Li et al. demonstrated a single GaAs_0.56_Sb_0.44_ nanowire photodetector with good responsivity and detectivity at a low operating bias voltage of 0.15 V at both 1.3 and 1.55 μm telecommunication wavelengths by tuning the bandgap of GaAsSb (Figure 1) [9]. InGaAs nanowires have also been reported as room temperature high-performance NIR photodetectors with high responsivity of 6.5 × 10^3^ A/W and *EQE* of 5.04 × 10^5^% [77]. Ren et al. synthesized InAs*_x_*P_1-*x*_ (0 ≤ *x* ≤ 1) nanowires that span the whole range of *x* and studied their photoresponse in the broad IR range from 0.7 to 3.5 µm [78]. A responsivity of 5.417 × 10^3^ A/W and *EQE* of 3.96 × 10^5^% at 1.7 µm and 0.5 V were demonstrated for InAs_0.52_P_0.48_ [78]. It was also found that the ternary InAs*_x_*P_1-*x*_ nanowire detectors showed better performance than binary InP (R of 337 A/W at 0.9 µm) and InAs (R of 1.668 × 10^3^ A/W at 2.9 µm) nanowires, presenting a highest value of R when *x* = 0.52 [78]. This improved performance was attributed to the fact that electron concentration *n* of InAs*_x_*P_1-*x*_ alloys was first increased and then decreased when *x* was increased from 0 to 1, showing the peak concentration at *x* = 0.52, whereas the electron mobility remained almost unchanged because alloy scattering is not dominant at room temperature, leading to higher electron density than binary nanowires [78,79].

### 3.2. Phototransistors

**Figure 2:**
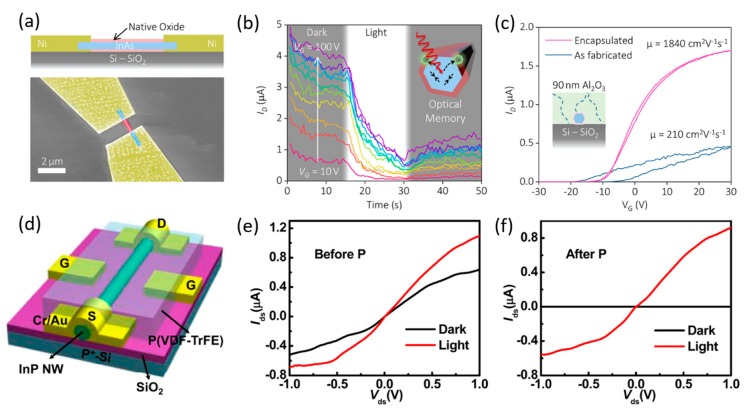
(**a**) Schematic of a back-gated single InAs nanowire phototransistor. (**b**) Time-dependent photoresponse of the device at various gate voltages. (**c**) Transfer curves of an InAs nanowire transistor before and after surface passivation of Al_2_O_3_, with a schematic shown in the inset indicating possible pinhole defects within the Al_2_O_3_ layer. Reproduced with permission [36]. Copyright 2017, American Chemical Society. (**d**) Schematic of a ferroelectric side-gated single InP nanowire photodetector and its *I*–*V* behavior (**e**) before and (**f**) after polarization. Reproduced with permission [35]. Copyright 2016, American Chemical Society.

Single horizontal nanowires are usually fabricated on thermally oxidized Si substrates with electrodes on the two ends of the nanowire for drain and source contacts, as shown in Figure 2a. When a gate voltage is applied to the back of the heavily doped Si substrate, a field effect transistor (FET) structure is formed [37]. Alternatively, applying an insulating layer and a contacting layer on top of the nanowire, a top-gated FET can be achieved as shown in Figure 2d [11,35]. Such transistor structures have been used for InAs photodetectors to manipulate electron trapping sites formed by the native oxide, leading to tunable positive or negative photoconductivity [11,36,37,80,81]. Although most semiconductors have increased conductivity under photoexcitation (positive photoconductivity (PPC)), Alexander-Webber et al. reported an unusual negative photoconductivity (NPC) of InAs nanowire-based phototransistors under visible light illumination as shown in Figure 2b [36]. This surface photo-gating effect may be explained by the fact [36,80] that the free carriers in the InAs nanowire could be excited into charge trapping sites located in the surface native oxide after the absorption of light with photon energy much higher than the bandgap. It was also found that the time-dependent photoresponse of the device at increased gate voltages showed a more obvious and gradually saturated NPC behavior under illumination and increased current after the light was switched off with an optical memory effect [36]. Surface passivation of the transistors with a 90 nm layer of Al_2_O_3_ can be used to significantly reduce charge trap density with an order of magnitude increase in field-effect mobility (*µ*), leading to a PPC in InAs nanowires [36]. Zheng et al. demonstrated a top-gated InP nanowire photodetector in which the dark current was significantly suppressed by applying an ultrahigh electrostatic field to polarized P(VDF-TrFE) ferroelectric polymer as shown in Figure 2d–f [35]. This behavior could be maintained even when the gate voltage was removed. Very high photoconductive gain of 4.2 × 10^5^, responsivity of 2.8 × 10^5^ A/W, and specific detectivity of 9.1 × 10^15^ Jones at *λ* = 0.83 µm were measured [35]. More recently, Zhang et al. developed a P(VDF-TrFE)-coated InAs nanowire photodetector that exhibited an ultrasensitive photoresponse in a wide spectral range extending to MWIR [81,82]. The electrostatic field of the polarized ferroelectric material was capable of modifying the surface electron–hole distribution and the InAs nanowire band structure, resulting in a photoresponse at a MWIR wavelength of 4.3 µm (well below the InAs band gap) and a responsivity of 9.6 × 10^3^ A/W and detectivity of ∼8.5 × 10^10^ Jones (cm·Hz^1/2^W^−1^) at 77 K [82].

Single InAs nanowire-based FETs have also been successfully developed as highly sensitive room temperature THz detectors with a detection frequency range from 0.3 to 2.8 THz [83,84,85,86]. As the active element, these FETs could work as rectifying diodes with modulated photoconductivity when the electromagnetic radiation was funneled onto a resonant or broadband antenna and excites plasma oscillations [85]. As InAs nanowires have a narrow bandgap and degenerate Fermi-level pinning, these devices are promising for bolometric detection with scaled down-dimensions when compared with planar structures [85]. In addition, these devices could be used in continuous-wave THz transmission imaging applications as demonstrated by Romeo et al. [85,86].

### 3.3. Junction Based Photodetectors

**Figure 3:**
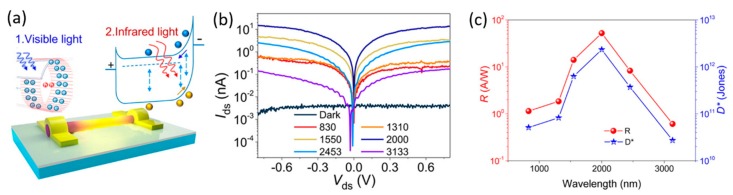
(**a**) Schematic of a single InAs infrared M-S-M photodetector and its working principle by using a visible light-assisted dark-current suppressing method. (**b**) Dark and light illumination *I*–*V* characteristics and (**c**) responsivity and detectivity spectra of the fabricated detector. Reproduced with permission [39]. Copyright 2016, American Chemical Society.

Fabrication of proper metal contacts or implementing doping can be used to form Schottky metal-semiconductor, homo- or heterojunction in nanowires for photodetection. A Schottky barrier tends to be formed when some noble metals such as Au and Ag make contact with a semiconductor such as InP and GaAs [87]. The junction at the interface between metal and semiconductor nanowire produces photocurrent with contributions from photoexcited electron–hole pairs in the semiconductor and electrons in the metal under reversed high electric field across the junction [32]. Due to the morphology and associated properties of the nanowires, the performance of these photodetectors could be tuned by adjusting the Schottky barrier height, which is sensitive to the carrier generation and transport [88]. As the photocurrent is normally localized near the metal electrode–nanowire contact, scanning photocurrent mapping can be an effective way to investigate the effects of Schottky barriers on the mechanisms of photoconduction [32,75,89]. Compared to the M-S structure-based Schottky barrier photodiode that can produce photocurrent at zero bias with a gain no more than 1 but very fast response time [32], the M-S-M structure is symmetrical at zero bias with a built-in potential well for photoexcited carriers until a high external voltage is applied to break through the potential barrier. Photodiodes based on the M-S-M structure have been shown to possess many desirable characteristics such as high speed, gain, and photosensitivity [32,38,60,88]. Thunich et al. used focused ion beam (FIB) deposition technique to form Au/GaAs Schottky junctions on a p-doped GaAs nanowire with a response time faster than 200 µs [38]. Dai et al. designed a GaAs/AlGaAs photodetector using an M-S-M radial architecture that formed built-in electric fields at the semiconductor hetero-interface and the metal/semiconductor Schottky contact to promote photogenerated charge separation and thus enhance the photosensitivity [44]. As shown in Figure 3, Fang et al. proposed a visible light-assisted dark-current suppressing method to enhance the barrier height of the metal–semiconductor contact and form an M-S-M photodiode. The dark current was reduced and the infrared photodetection was broadened from 0.83 to 3.133 µm with a high responsivity of 40 A/W, detectivity of ∼10^10^ Jones, and fast response time of 80 µs at *λ* = 2 µm at a low bias voltage of 0.1 V [39]. Kuo et al. fabricated single InSb nanowires based on M-S-M structure (Pt-InSb-Pt) as MWIR photodetectors and demonstrated a superior responsivity of 8.4 × 10^4^ A/W at MWIR wavelength of 5.5 µm [74].

**Figure 4:**
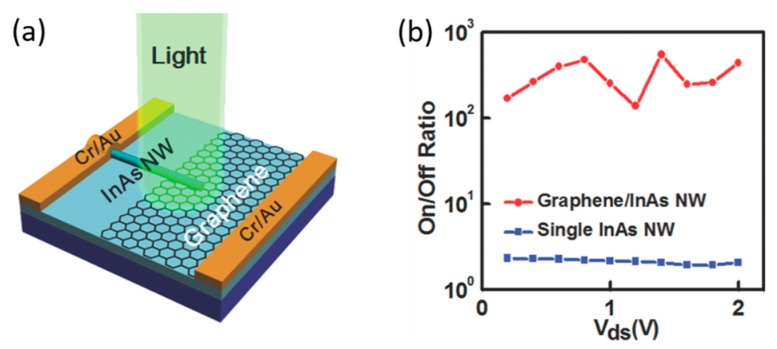
(**a**) Schematic and (**b**) *I*_light_ /*I*_dark_ ratios of a graphene/InAs nanowire heterojunction infrared photodetector with much enhanced *I*_light_ /*I*_dark_ ratio in comparison with the single horizontal InAs nanowire photodetectors. Reproduced with permission [45]. Copyright 2014, Wiley-VCH Verlag GmbH & Co. KGaA, Weinheim.

In addition to the aforementioned configurations, p-n or p-i-n nanowire photodiodes have been employed to operate under photovoltaic mode at zero bias [33] or photoconductive mode at reverse biases. Homojunctions can be formed either axially along the nanowire or radially conforming the nanowire core by a shell [33], whereas heterojunctions are normally formed by the core–shell configuration or a nanowire vertically grown on a substrate with different materials. Such junctions showed rectifying behavior to suppress the dark current and enhance the detector’s sensitivity, with relatively fast photoresponse [32]. Brenneis et al. demonstrated a Si/InAs heterojunction infrared photodetector formed by a single vertical unintentionally n-type InAs nanowire grown on a p-type Si substrate [43]. Miao et al. presented a graphene/InAs nanowire heterojunction photodetector with an *I*_light_/*I*_dark_ ratio of 500 at *λ* = 1 µm by integrating a horizontal single InAs nanowire on a vertically stacked graphene layer as shown in Figure 4 [45]. Ma et al. reported a GaSb/GaInSb p-n heterojunction based photodetector [42]. High responsivity of 10^3^ A/W, *EQE* of 10^4^ and response time of 2 ms at the optical communication wavelength of 1.55 µm were obtained by applying a high forward bias (+1 V). Wang et al. demonstrated a GaAsSb/InAs p-n heterojunction-based photodetector that showed a wide spectral photoresponse ranging from 0.488 to 1.8 µm [90]. A responsivity of 0.0046 and 0.12 A/W to 1.31 µm telecommunication light was obtained under −0.3 and +0.3 V, respectively [90].

When a photodiode operates as an avalanche photodetector (APD) at a high reverse bias condition, impact ionization leads to multiplication of holes and electrons created by the initial photon excitation, enabling large photocurrent gain and sensitivity. The intrinsic small photon collection area and active volume of nanowire-based APDs make them especially attractive device structures to enable photocurrent gain and sensitivity even down to the single photon level. Linear mode APDs have been reported in ensemble InGaAs/GaAs core–shell nanoneedles [91], exhibiting extremely high gain values at bias voltages significantly below breakdown [92] due to the reduction of active material volume. More significantly, a vertical InGaAs/GaAs nanowire array separate absorption-multiplication (SAM) APD operating in Geiger mode has also been demonstrated recently [93]. The nanowire array contained thousands of nanowires with each avalanche event confined in a single nanowire, drastically reducing the avalanche volume and the number of filled traps. This led to an extremely small afterpulsing probability compared with conventional InGaAs-based single-photon avalanche diodes (SPADs) and enabled operation in free-running mode up to 150 K, which is attractive for emerging integrated photonics and quantum information applications [93].

### 3.4. Photoconductive Switch THz Detectors

**Figure 5:**
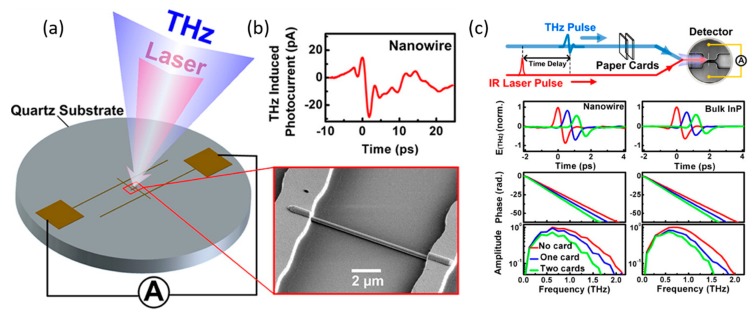
(**a**) Schematic of the detector design and optical arrangement of a single GaAs/AlGaAs/GaAs core–shell–cap nanowire photoconductive THz detector and SEM image of the central area of a fabricated device. (**b**) Time-domain THz response of the detector characterized in a THz-TDS system. (**c**) Schematic of the THz signal transmission measurement in a THz-TDS system when paper cards are presented. Reproduced with permission [94]. Copyright 2014, American Chemical Society.

GaAs [94,95] and InP [63,96] nanowires have also been explored as photoconductive terahertz (THz) detectors, as a promising alternative to bulk semiconductor detectors due to its nanoscale spatial resolution and intrinsic polarization-resolved sensitivity. A single-nanowire photoconductive antenna (SNW-PCA) can be integrated into a pulsed THz time-domain spectroscopy (TDS) system with a Ti:sapphire laser to obtain the time-domain THz response (THz induced photocurrent) of a nanowire detector as shown in Figure 5a,b [94]. Kun et al. successfully demonstrated a narrow bandwidth single GaAs/AlGaAs/GaAs core–shell-cap nanowire photoconductive THz detector that operated well in the range of 0.1 to 0.6 THz due to local field enhancement by a simple two-pad antenna [94]. Further optimization of the antenna design yielded a single InP nanowire photoconductive detector with a high-amplitude and phase-sensitive time-domain spectrum in a broad bandwidth range of 0.1 to 2 THz [96]. The contact quality was improved using an n^+^-i-n^+^ structure implemented for the single InP nanowire THz detectors, leading to enhancement of signal-to-noise ratio, ~2.5 times of the undoped InP nanowire detector [63]. The function of the single nanowire THz detector has also been further confirmed by measuring the absorption coefficient and refractive index of paper cards in a THz-TDS system where a single InP nanowire detector was used to measure the transmitted THz signal through the paper card as shown in Figure 5c [96].

### 3.5. Hybrid-Type Nanowire Detectors

**Figure 6:**
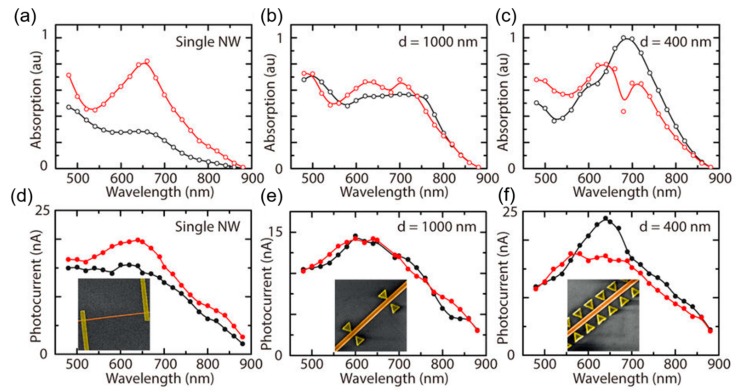
(**a**,**b**,**c**) Simulated absorption spectra and (**d**,**e**,**f**) measured photocurrent spectra of single nanowire and hybrid nanowire/nanoantenna structures for light perpendicular to the nanowire axis (black) and parallel to the nanowire axis (red), respectively. Reproduced with permission [48].

Plasmonic structures could be combined with nanowires to enhance the detector performance and polarization sensitivity. Casadei et al. demonstrated a nanowire/nanoantenna hybrid structure that could be used to engineer the polarization response of nanowires by embedding a GaAs horizontal nanowire in a bow-tie antenna array, as shown in Figure 6 [48]. The nanowire coupled with the plasmonic modes of the bow-tie nanoantennas when excited with transverse polarized light (perpendicular to the nanowire axis), thus enhancing light absorption (Figure 6a–c) and polarization photoresponse (Figure 6d–f) [48]. Senanayake et al. successfully fabricated self-aligned metal nanoholes on vertical nanopillars as a 2-dimensional (2D) plasmonic crystal to realize plasmon-enhanced photodetectors that could tune the peak responsivity wavelength and polarization with enhanced optical coupling efficiency [17,97].

Table 1 presents a summary of the key performance metrics of the reported III-V single nanowire infrared photodetectors measured at room temperature.

## 4. Challenges and Future Perspectives

Although III-V semiconductor nanowires present unique and excellent optical and electrical properties and have been demonstrated to have promising performance for room temperature infrared photodetection, there are still many technological challenges to overcome. Despite good device performance, the output current of the single nanowire photodetectors is still too low for practical applications. Therefore, the fabrication technique for large-scale and controlled assembly of single nanowires into horizontal arrays is highly desirable [102]. There have been a few techniques reported for nanowire position alignment, such as optical tweezers [103], electric field-assisted assembly technique [102,104], elastomeric poly(dimethylsiloxane) (PDMS) stamp-assisted transfer [105], Langmuir–Blodgett method [106], bespoke polymer μ-stamp-assisted nano-scale transfer printing technique [107,108], as well as a large-scale transfer printing technique known as contact printing [53,109], by simply transferring nanowires from the growth substrate to a patterned receiver substrate. However, there is always a trade-off between the accuracy of nanowire positioning and scalability of the technique. This requires further development of the technology for high-performance, stable-operation, and low-cost assembly of horizontal nanowire arrays on both rigid and flexible substrates.

Another limitation of III-V semiconductor nanowire photodetectors is their detection wavelength that are determined by the material bandgap, especially for the relatively easy synthesis but wider bandgap binary materials such as GaAs and InP. To enable light detection of photon energies well below the semiconductor bandgap, Knight et al. proposed a novel idea of active optical antennas, in which hot carriers are generated and injected into the semiconductor material (e.g., Si substrate) through the antenna–semiconductor Schottky barrier, contributing to a detectable photocurrent response at SWIR wavelengths up to 1.6 μm [110]. To further extend the nanowire photodetector photoresponse range to long-wavelength infrared (LWIR), 0-dimensional (0D) nanomaterials, such as semiconductor quantum dots (QDs) and quantum wells (QWs), could be incorporated into nanowire structures. Synthesis of single or multiple low-bandgap InAsP quantum discs (QDiscs) within an InP nanowire axially was demonstrated by Karimi et al. such that the intersubband transitions in the conduction band of the InAsP QDiscs enabled broad infrared response ranging from 3 to 20 μm [111].

## 5. Conclusions

Single III-V semiconductor nanowires have been extensively explored in the past decade as room temperature high-performance infrared photodetectors with broad detection wavelength range covering UV, VIS, IR, and THz regimes. In this article, we present a review on their recent development in materials, structures, and comparative performances, as well as the existing challenges and possible future directions.

## Figures and Tables

**Table 1:** Summary of the key performance metrics of room temperature III-V single nanowire infrared photodetectors.

NW Material	Band Gap (eV)	Growth Details	Device Structure	*λ* (µm)	*R* (A/W)	D* (Jones)	*G*	*EQE* (%)	*t* (µs)	Ref.
InP	1.338 (zincblende (ZB)) and 1.408 (wurtzite (WZ))	Bottom-up, metal organic CVD (MOCVD), Au-assisted VLS	Horizontal, M-S-M Schottky photodiode	0.7–1						[98]
InP	1.34	CVD	Horizontal, phototransistor with polarization ofP(VDF-TrFE) ferroelectric polymer	0.5–1.2	2.8 × 10^5^@0.83 µm, 1 V	9.1 × 10^15^	4.2 × 10^5^		2.91 × 10^4^	[35]
InP	1.42	Bottom-up, MOCVD, SAE	Horizontal, photoconductor	0.1–2 THz						[96]
InP	1.41	Bottom-up, MOCVD, SAE	Horizontal, n^+^-i-n^+^ photoconductor	0.1–2.2 THz						[63]
GaAs	1.42	Bottom-up, MBE, Ga-assisted VLS	Horizontal, Schottky photodiode	0.7–1					167	[38]
GaAs	1.42	Bottom-up, MOCVD, Au-assisted VLS	Horizontal, M-S-M Schottky photodiode	0.4–1.2			2 × 10^4^			[88]
GaAs	1.51 for undoped NW@77 K; 1.45 for 2 × 10^17^ cm^−3^ n-doped NW@77 K	Bottom-up, MBE	Vertical on GaAs substrate, Schottky photodiode	0.808	5.3 ×10^−7^@10 V for undoped NW; 1.5 × 10^−7^@10 V for 2 × 10^17^ cm^−3^ n-doped NW				1 × 10^−6^	[66]
i-GaAs/p-GaAs core–shell		Bottom-up	Horizontal, photoconductor with electrical contact on p-type shell	0.48–0.88						[48]
GaAs/ AlGaAs core–shell		Bottom-up, MOCVD, Au-assisted VLS	Horizontal, M-S-M Schottky photodiode	0.8	10^−4^			13.5	5 × 10^−3^	[71]
GaAs/ AlGaAs core–shell		Bottom-up, MOCVD, Au-assisted VLS	Horizontal, M-S-M Schottky photodiode	0.5–0.9						[89]
GaAs/ hight-T GaAs/ AlGaAs core-multi shell	1.412	Bottom-up, MOCVD, Au-assisted VLS	Horizontal, radial heterojunction M-S-M Schottky photodiode	0.3–0.9	0.57@0.855 µm, 1 V	7.20 × 10^10^				[44]
GaAs/ AlGaAs/GaAs core–shell-cap	1.43	Bottom-up, MOCVD, Au-assisted VLS	Horizontal, photoconductor	0.1–0.6 THz					4.6 × 10^−3^	[94]
GaAs/ AlGaAs/GaAs core–shell-cap	1.43	Bottom-up, MOCVD, Au-assisted VLS	Horizontal, photoconductor	0.1–1.5 THz					4.6 × 10^−3^	[95]
GaSb	0.725	Horizontal tube furnace, CVD	Horizontal, photoconductor	0.35–0.8	443.3@0.8 µm, 0.4 V	2.86 × 10^9^@ 0.8 µm		688.4@0.8 µm	2 × 10^5^@0.6 µm	[73]
GaSb/ GaInSb	0.75 for GaSb@77 K; 0.625 for Ga_0.9_ In_0.1_ Sb@77 K	Horizontal tube furnace, CVD	Horizontal, p-n heterojunction photodiode	0.78–2.25	1.5 × 10^3^@1.55 µm, 1 V			8.5 × 10^6^	2 × 10^3^	[42]
InAs	0.354	Bottom-up, CVD, Au-assisted	Horizontal, photoconductor & phototransistor	0.3–1.1	4.4 × 10^3^@ 0.532 µm, 15 V	2.6 × 10^11^		1.03 × 10^6^		[99]
InAs	0.365	Bottom-up, MBE, Au-assisted	Horizontal, photoconductor Schottky photodiode & phototransistor	0.632–1.47	5.3 × 10^3^ for photodiode; 1.9 × 10^3^ for photoconductor					[11]
InAs	0.35	Bottom-up, MBE, Au-assisted	Horizontal, photoconductor & phototransistor	0.5–1.6	−3 × 10^4^@0.2 V		−7.5 × 10^4^		<100	[80]
InAs	0.35	Bottom-up, MBE, Au-assisted VLS	Horizontal, M-S-M Schottky photodiode, VIS light (450 nm)-assisted	0.83–3.133	40@2 µm, 0.1 V; 0.6@3.113 µm, 0.4 V	2 × 10^12^@2 µm; 10^10^@3.113 µm			80	[39]
InAs		Bottom-up, MOCVD, self-assisted	Horizontal, phototransistor	0.633, 0.65–1					<2.5 × 10^5^	[37]
InAs	0.477	Bottom-up, MOCVD, Au-assisted VLS	Horizontal, phototransistor	halogen lamp with a peak@ 0.906 µm (1.37 eV)						[36]
InAs		Bottom-up, chemical beam epitaxy (CBE), Au-assisted VLS	Horizontal, phototransistor	0.3 THz	>1 V/W@V_gs_ = −7 V and V_ds_ = 0.01 V					[83]
InAs		Bottom-up, CBE, Au-assisted VLS	Horizontal, phototransistor	1.5 THz	12 V/W@V_gs_ = -2 V and V_ds_ = 0.005 V					[84]
InAs		Bottom-up, CBE, Au-assisted VLS	Horizontal, phototransistor	0.3 THz	100 V/W@V_gs_ = 5 V					[85]
InAs		Bottom-up, CBE, Au-assisted VLS	Horizontal, phototransistor	2.8 THz	5 V/W@V_gs_ = 8 V and V_ds_ = 0.025 V					[86]
InAs/Si	0.36	Bottom-up, MBE, SAE	Vertical InAs NW on p-type Si substrate, p-n heterojunction photodiode	1.47						[43]
InAs/ Graphene		Bottom-up, MBE, Au-assisted	Horizontal, hetero junction photodetector	0.457–1	0.5					[45]
InSb	0.18	Bottom-up, direct current (DC) electro deposition with AAO template	Horizontal, photoconductor & M-S-M Schottky photodiode	0.5–1						[100]
InSb	0.17–0.4	Bottom-up, tube furnace, Au-assisted VLS	Horizontal, Schottky photodiode	1000 °C black body radiation				60		[41]
InSb	0.17	Bottom-up, electrochemical method with AAO template	Horizontal, M-S-M Schottky photodiode	5.5	8.4 × 10^4^			1.96 × 10^6^	2.6 × 10^5^	[74]
InAsP	1.416(InP)–0.421 (InAs)	VLS and ion-exchange (IE)	Horizontal, photoconductor	0.7–3.5	1668@2.9 µm, 0.5 V for InAs; 4998@2.3 µm for InAs_0.8_P_0.2_; 5417@1.7 µm, 0.5 V for InAs_0.52_P_0.48_; 3833@1.2 µm, 0.5 V for InAs_0.25_P_0.75_; 337@0.9 µm, 0.5 V for InP			7.15 × 10^4^ for InAs; 2.7 × 10^5^ for InAs_0.8_ P_0.2_; 3.96 × 10^5^ for InAs_0.52_ P_0.48_; 3.67 × 10^5^ for InAs_0.25_ P_0.75_; 4.65 × 10^4^ for InP		[78]
InGaAs	0.69@77 K for In_0.65_Ga_0.35_As	Bottom-up, CVD, Au-assisted	Horizontal, photoconductor	1.1–2	6.5 × 10^3^@1.6 µm, 0.5 V			5.04 × 10^5^	7 × 10^4^	[77]
GaAsSb	0.9@77 K for Ga As_0.26_Sb_0.74_	CVD	Horizontal, photoconductor	1.16–1.55	1.7 × 10^3^@1.31 µm, 1 V			1.62 × 10^5^	6 × 10^4^	[101]
GaAsSb	0.827 for Ga As_0.56_Sb_0.44_	Bottom-up, MOCVD, Au-assisted VLS	Horizontal, photoconductor	1.1–1.66	2.37@1.3 µm, 0.15 V; 1.44@1.55 µm, 0.15 V	1.08 × 10^9^@ 1.3 µm; 6.55 × 10^8^@ 1.55 µm				[9]
GaAsSb/InAs	1.1 for Ga As_0.82_Sb_0.18_	Bottom-up, solid-source MBE, self-assisted VLS	Horizontal, p-n heterojunction photodiode	0.488–1.8	0.12@1.31 µm, 0.3 V			12	4.5 × 10^2^@0.633 µm	[90]
GaAsSb/InP	0.92 for As_0.66_Sb_0.34_	Bottom-up, MOCVD, Au-assisted VLS	Horizontal, photoconductor	1.05–1.55	143.5@1.3 µm, 1.5 V for GaAsSb core-only; 325.1@1.3 µm, 1.5 V for GaAsSb/InP core–shell	5.3 × 10^10^@1.3 µm, 1.5 V for GaAsSb core-only; 4.7 × 10^10^@1.3 µm, 1.5 V for GaAsSb/InP core–shell		1.37 × 10^4^ for GaAsSb core-only; 3.1 × 10^4^ for GaAsSb/InP core–shell;		[76]
